# A Novel Unsupervised Structural Damage Detection Method Based on TCN-GAT Autoencoder

**DOI:** 10.3390/s25216724

**Published:** 2025-11-03

**Authors:** Yanchun Ni, Qiyuan Jin, Rui Hu

**Affiliations:** 1 College of Civil Engineering, Tongji University, Shanghai 200092, China; yanchunni@tongji.edu.cn (Y.N.); 2330880@tongji.edu.cn (Q.J.); 2 Guangdong Provincial Key Laboratory of Intelligent and Resilient Structures for Civil Engineering, Harbin Institute of Technology, Shenzhen 518055, China

**Keywords:** damage detection, multi-sensor, structural health monitoring, unsupervised deep learning, temporal convolutional network, graph attention network

## Abstract

Over the service life of several decades, structural damage detection is crucial for ensuring the safety and durability of engineering structures. However, existing methods often overlook the spatiotemporal coupling in multi-sensor data, hindering the full exploitation of structural dynamic evolution and spatial correlations. This paper proposes an autoencoder model integrating Temporal Convolutional Networks (TCN) and Graph Attention Networks (GAT), termed TCNGAT-AE, to establish an unsupervised damage detection method. The model utilizes the TCN module to extract temporal dependencies and dynamic features from vibration signals, while leveraging the GAT module to explicitly capture the spatial topological relationships within the sensor network, thereby achieving deep fusion of spatiotemporal features. The proposed method adopts an “offline training-online detection” framework, requiring only data from the healthy state of the structure for training, and employs reconstruction error as the damage indicator. To validate the proposed method, two sets of experimentally measured data are utilized: one from the Z-24 concrete box-girder bridge under ambient excitation, and the other from the Old Ada Bridge under vehicle load excitation. Additionally, ablation studies are conducted to analyze the effectiveness of the spatiotemporal fusion mechanism. Results demonstrate that the proposed method achieves effective damage detection in both different structural types and excitation scenarios. Furthermore, the explicit modeling of spatiotemporal features significantly enhances detection performance, with the anomaly detection rate showing substantial improvement compared to baseline models utilizing only temporal or spatial modeling. Moreover, this end-to-end framework processes raw vibration signals directly, avoiding complex preprocessing. This makes it highly suitable for practical and near-real-time monitoring. The findings of this study demonstrate that the damage detection method based on TCNGAT-AE can be effectively applied to structural safety monitoring in complex engineering environments, and can be further integrated with real-time monitoring systems of critical structures for online analysis.

## 1. Introduction

As modern civil engineering structures grow in scale and service life, Structural Health Monitoring (SHM) has become pivotal for ensuring safety and durability, 200 attracting significant academic and engineering interest [1]. The primary objective of SHM systems is the timely assessment of structural conditions, with effective damage diagnosis constituting its core function. Throughout the long-term service life of structures, factors including external loading, environmental fluctuations, and material degradation inevitably lead to damage accumulation. Such damage not only compromises structural load-bearing capacity but may also pose substantial threats to personnel and property safety. Thus, developing efficient and reliable damage identification methods remains a key challenge in SHM.

Vibration-based damage identification methods, recognized for their non-destructive nature, cost-effectiveness, and sensitivity to global damage, have become one of the most extensively utilized techniques in SHM [2]. Recent advancements in sensor technology, data acquisition systems, computational capabilities, and particularly deep learning, have substantially enhanced the diagnostic performance of these methods [3,4]. Deep learning algorithms demonstrate proficiency in processing massive vibration datasets and extracting latent features, exhibiting prominent advantages in complex structural systems and high-noise environments. Consequently, this technology has not only achieved considerable progress in scientific research but also demonstrated significant application potential in practical engineering, emerging as a crucial approach for ensuring the operational safety of large-scale civil infrastructures including bridges, long-span structures, and high-rise buildings [5,6].

A Convolutional Neural Network (CNN), as a representative deep learning architecture, has been widely employed for structural damage identification. Lin et al. [7] proposed a deep CNN-based approach capable of autonomously extracting damage-sensitive features from sensor data, enabling accurate structural damage localization without manual feature engineering, thereby overcoming the dependency on handcrafted features inherent in conventional methods. To further enhance model performance, subsequent research has implemented various modifications to CNN architectures. Abdeljaber et al. [8] applied a 1D-CNN to steel frame damage detection; Dang et al. [9] developed a four-branch 1D-CNN architecture; Zhang et al. [10] constructed a streamlined 1D-CNN to detect minor variations in local stiffness and mass within actual structures, validating its high sensitivity to subtle state changes; Shang et al. [11] employed CNNs to establish a deep denoising autoencoder, achieving unsupervised detection of minor damage.

However, the convolutional operations in CNNs are inherently local perception processes, limiting their capacity to capture long-range temporal dependencies [12,13]. To address this limitation, neural networks including Long Short-Term Memory (LSTM) and Temporal Convolutional Networks (TCN) have been introduced for temporal modeling of vibration signals. For instance, Chen et al. [14] developed a 1DCNN-BiLSTM deep learning model capable of detecting localized minor structural changes in reinforced concrete beams while maintaining high accuracy under noisy conditions. Sony et al. [15] constructed a supervised LSTM model, validated through experimental data and Z24 bridge benchmark data, demonstrating performance superior to 1DCNN. Yessoufou et al. [16] implemented a composite encoder–decoder network based on LSTM, enabling rapid unsupervised assessment of bridge damage. Ghazimoghadam et al. [17] proposed a multi-head self-attentive LSTM autoencoder that integrates multi-sensor information for precise damage localization and quantification. Hasani et al. [18] proposed an AI-driven automated integrated SHM framework, within which discrete wavelet transform was integrated with an Autoencoder-LSTM network to achieve structural damage localization. Li et al. [19] proposed a method leveraging a TCN and a multi-branch hybrid attention residual network to capture long-term temporal dependencies for bearing fault diagnosis. Gu et al. [20] presented a supervised framework whose efficacy primarily stems from the exceptional temporal modeling capability of a Bidirectional TCN, which comprehensively extracts contextual signal features, achieving nearly perfect accuracy in imbalanced blade damage identification.

Although models such as LSTM can effectively characterize temporal patterns in vibration data, they often fail to utilize the physical spatial information embedded in sensor networks, while structural damage initiation and evolution exhibit significant spatial correlations. Graph Neural Networks (GNNs) overcome the limitation of traditional neural networks being unable to explicitly model physical spatial relationships among multiple sensors, and are progressively being applied in the SHM domain [21,22]. Dang et al. [23] proposed a supervised framework integrating 1D-CNN and GNN, representing the sensor network as a graph structure to enhance damage detection accuracy. To further improve classification performance, Dang et al. [24] subsequently incorporated LSTM to construct a spatiotemporal network model, while also introducing a semi-supervised damage detection method combining GNN with contrastive learning that requires minimal labeled data for training [25]. Kim et al. [26] developed a near-real-time seismic damage identification method based on a bifurcated Graph Convolutional Autoencoder (GCAE), employing a mode shape-based adjacency matrix to account for spatial correlations; in subsequent research [27], they further established a dynamic GNN based on Proper Orthogonal Decomposition for near-real-time damage identification. Miele et al. [28] designed an unsupervised anomaly detection framework for wind turbine towers, constructing dynamic graph structures based on mutual information and utilizing Graph Autoencoders to capture spatial features. Kuo et al. [29] proposed a GNN-LSTM model for structural dynamic response prediction through joint capture of spatiotemporal information. Regarding attention mechanisms. Zhao et al. [30] proposed the Exponential Smoothing Multi-Head Graph Attention Network (ESMGAT) method to address challenges such as single-sensor deployment and achieving precise damage zone localization in noisy environments. Furthermore, GAT demonstrates potential in multi-sensor information fusion. Meng et al. [31] proposed a multi-sensor fusion methodology based on Modal Analysis and Graph Attention Network (MFMAGAT) for supervised bearing fault diagnosis. Wang et al. [32] proposed a self-weighted graph attention network based on motor stator current signals, which enables effective fault diagnosis under extreme data imbalance.

In existing research methodologies, conventional deep learning models (e.g., CNN, LSTM) frequently neglect spatial topological relationships among sensors, while graph-based approaches (e.g., GAT), although effective in capturing spatial interactions, often inadequately model complex temporal dependencies in vibration signals. To concurrently address these limitations in temporal and spatial feature extraction, this paper proposes an unsupervised spatiotemporal modeling framework (TCNGAT-AE) that integrates TCN and GAT in a sequential architecture. The core concept involves the TCN module initially extracting temporal features from vibration signals from individual sensors, generating node features with dynamic evolution information. The GAT module subsequently constructs a graph structure based on sensor spatial topology and employs attention mechanisms to model spatial relationships among node features, thereby identifying damage-induced spatial mode variations. The entire model is structured as an autoencoder, trained exclusively on healthy state data, ultimately achieving precise and robust damage detection through reconstruction error.

The paper is organized as follows: Section 2 presents the theoretical foundations of TCN, GAT, and autoencoders; Section 3 provides a comprehensive description of the proposed methodology; Section 4 verifies the approach using two field test cases—first with Z-24 bridge data under vehicle load excitation, then with steel truss bridge data under vehicle loading—along with ablation experiments and in-depth result discussion; Section 5 summarizes the main findings and confirms the method’s effectiveness; Section 6 outlines future research directions.

## 2. Theoretical Basis

### 2.1. Temporal Convolutional Network

The TCN was introduced by Bai et al. [33] in 2018. Its core concept lies in integrating causal convolutions with dilated convolutions to equip the model with exceptional temporal modeling capabilities. A key characteristic of TCN is its strict causal constraint, implemented through causal convolutions. By employing unilateral zero-padding, causal convolutions ensure that the output at any given timestep depends solely on current and past inputs, completely preventing future information leakage and thereby fulfilling the causality requirement for temporal modeling.

Given a one-dimensional temporal signal, this operation can be formulated as:

(1) $$y_{t}=\sum_{i=0}^{k-1}w_{i}\cdot x_{t-i}$$

where $y_{t}$ is the output value at timestep t; $w_{i}$ is the i−th weight parameter of the causal convolution kernel; $x_{t-i}$ is the input value at timestep t−i; k is the kernel size of the causal convolution (specifying how many historical timesteps of inputs contribute to computing $y_{t}$; T is the temporal length of the input signal; and when t<k, $x_{t-k}=0$, ensuring the output is computed exclusively from historical data.

To effectively capture long-range dependencies, TCN incorporates dilated convolutions to exponentially expand the receptive field without significantly increasing the number of parameters or computational complexity. For a dilation factor d, the temporal span covered by the effective receptive field of the convolution kernel is calculated as:

(2) $$k_{effective}=k+(k-1)\times (d-1)$$

This mechanism enables TCN to capture both local details and global trends simultaneously, overcoming the limited receptive field bottleneck of traditional Convolutional Neural Networks in long-sequence modeling.

Compared to Recurrent Neural Networks and their variants (e.g., LSTM), TCN demonstrates higher stability during training, effectively mitigating gradient vanishing and explosion problems, thereby enhancing the model’s convergence speed and robustness. Leveraging the synergistic design of causality and dilated convolutions, TCN provides an efficient and reliable solution for temporal data analysis and has demonstrated outstanding performance in numerous tasks [20,33].

### 2.2. Graph Attention Network

**Figure 1:**
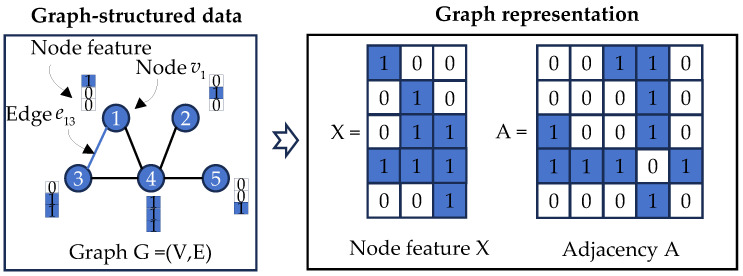
Illustrative example of graph representation.

A graph is a non-Euclidean data structure extensively employed to represent complex inter-entity relationships in real-world systems, including social networks, molecular structures, and sensor arrays. Mathematically, a graph is formally defined as G=(V,E), where V represents the vertex set comprising N nodes, with each node $v_{i}$ corresponding to a discrete entity or object; E denotes the edge set, characterizing interconnection relationships between nodes. For computational processing, graph structures are typically encoded through a node feature matrix $X\in {\mathbb{R}}^{N\times F}$, encapsulating original nodal attributes (where F indicates feature dimensionality), and an adjacency matrix $X\in {\mathbb{R}}^{N\times F}$, where entry $A_{ij}$ quantifies the edge existence between node pairs. Figure 1 demonstrates a representative graph structure containing five nodes.

The Graph Attention Network, introduced by Veličković et al. [34] in 2017, constitutes a graph neural network architecture based on spatial aggregation paradigm. Its fundamental innovation lies in incorporating attention mechanisms into graph node feature learning, effectively overcoming the limitation of conventional Graph Convolutional Network (GCN) that rely on static weight distributions during neighborhood aggregation, thereby enabling differentiated modeling of distinct neighbor nodes.

GAT implements dynamic feature aggregation through node-level attention mechanisms. The aggregation process is formulated as:

(3) $$z_{i}=ReLU\left(\alpha_{i,i}Wx_{i}+\sum_{j\in N(i)}\alpha_{i,j}Wx_{j}\right)$$

(4) $$g_{i}^{\left(t\right)}=Wx_{i}$$

(5) $$\pi (i,j)=LeakyReLU(a^{T}(g_{i}⊕g_{j}))$$

(6) $$\alpha (i,j)=softmax_{j}(\pi (i,j))=\frac{\exp\left(\pi (i,j)\right)}{\sum_{k\in N(i)\cup \left\{i\right\}}exp{\left(\pi (i,k)\right)}^{\prime }}$$

where $x_{i}$ represents the input feature vector of node i, W denotes the trainable weight matrix, and $\alpha_{ij}$ indicates the attention coefficient, and LeakyReLU, ReLU, and softmax are activation functions [35].

### 2.3. Autoencoder Architecture

**Figure 2:**
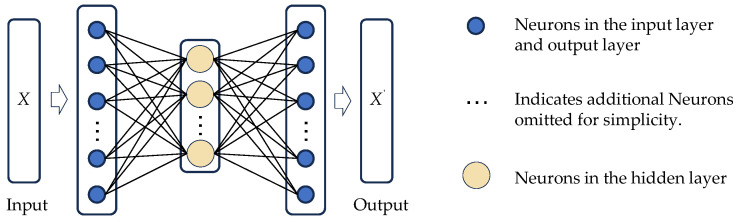
Illustrative example of Autoencoder architecture.

Autoencoders represent a classical type of unsupervised neural network model, characterized by a fundamental architecture comprising an encoder and a decoder. The encoder functions to map high-dimensional input data into a low-dimensional latent space, thereby producing a compact feature representation. The decoder subsequently reconstructs this latent representation into output matching the original input dimensions. Conventional autoencoders typically employ symmetrical encoder–decoder structures and are trained via minimization of reconstruction error (e.g., Mean Squared Error) between input and output. These models have demonstrated robust performance in applications including feature extraction, data dimensionality reduction, and anomaly detection. A schematic illustration of the autoencoder architecture is presented in Figure 2.

To enhance the flexibility and representational capacity of autoencoders in handling complex tasks, an effective strategy involves constructing heterogeneous encoder–decoder architectures. In such configurations, the encoder is typically deepened to learn more comprehensive hierarchical features, while the decoder is simplified to focus on efficient reconstruction. This design paradigm has demonstrated significant effectiveness in applications such as high-dimensional data anomaly detection [36,37,38].

## 3. Methodology

### 3.1. TCNGAT-AE Architecture

**Figure 3:**
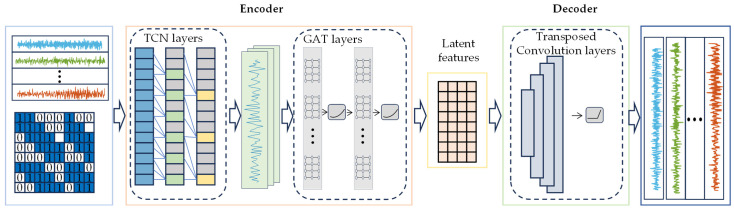
TCNGAT-AE architecture.

This paper proposes a hybrid deep learning model, termed Temporal Convolutional Graph Attention Autoencoder (TCNGAT-AE), for structural damage detection using multi-sensor temporal data. The model’s computational process involves three core stages (illustrated in Figure 3): First, the TCN module employs dilated convolutions and residual connections to extract dynamic features from vibration signals of individual sensors while effectively capturing long-range temporal dependencies. Next, these TCN-derived features serve as node inputs to construct a graph structure based on the physical configuration of the sensor network. A GAT is then applied to explicitly model spatial correlations among sensors through attention mechanisms, enabling deep fusion of multi-source features. Finally, a decoder comprising transposed convolutional layers reconstructs the integrated spatiotemporal features, with model training conducted in an unsupervised manner by minimizing the reconstruction error between input and output.

The model’s core innovation lies in its cascaded TCN-GAT architecture that facilitates explicit and synergistic modeling of spatiotemporal characteristics in vibration data. This approach effectively addresses the common limitation in conventional methods where temporal and spatial information processing remains segregated, thereby producing more discriminative feature representations for data-driven damage detection under complex operational conditions.

#### 3.1.1. Encoder

The encoder extracts spatiotemporal features through two cascaded modules: one for temporal feature extraction and another for spatial correlation modeling. The temporal modeling module comprises multiple stacked TCN layers that systematically expand the receptive field by progressively increasing dilation rates across layers. This architecture enables hierarchical capture of temporal patterns ranging from local dynamics to global evolutionary trends while maintaining the independence of individual sensor channels. The spatial modeling module constructs a graph structure based on the physical sensor topology, utilizing the high-level temporal features generated by the TCN as node attributes. Through a GAT, it adaptively learns attention weights for inter-node connections, ultimately producing a compact feature representation that integrates spatiotemporal contextual information from multiple sensors.

#### 3.1.2. Decoder

The decoder is responsible for reconstructing the original input signals from the bottleneck features generated by the encoder. This module utilizes multiple transposed convolutional layers for upsampling, with carefully tuned kernel parameters to progressively recover the temporal length. To improve training stability, each transposed convolutional layer is followed sequentially by Batch Normalization (BN) and ReLU activation, which effectively stabilizes gradient flow while enhancing feature representation capacity.

#### 3.1.3. Loss Function

During the model training phase, the optimization objective is set to minimize the Mean Squared Error (MSE) between input and output sequences. All trainable parameters in both the encoder and decoder are iteratively updated through the backpropagation algorithm, ensuring the model systematically minimizes the deviation between original inputs and their reconstructed counterparts. Notably, the MSE metric serves a dual purpose as the fundamental indicator for damage detection: under structural healthy conditions, the MSE values follow a stable statistical distribution, whereas the emergence of structural damage causes localized MSE values corresponding to anomalous sensors to exhibit marked deviation from the established healthy pattern. The complete TCNGAT-AE architecture is schematically presented in Figure 3.

The MSE loss function is formally defined as:

(7) $$l=MSE=\frac{1}{N}\sum_{i=1}^{N}\left({\left\|x_{i}-{\tilde{x}}_{i}\right\|}^{2}\right)$$

where N denotes the total number of samples in a training batch, $x_{i}\in {\mathbb{R}}^{C\times T}$ denotes the original input data of the i−th sample, where C is the number of sensors and T is the length of the temporal window; ${\tilde{x}}_{i}\in {\mathbb{R}}^{C\times T}$ represents the reconstructed output data of the i−th sample.

### 3.2. Damage Detection Process Based on TCNGAT-AE

This paper proposes an unsupervised damage detection framework model based on TCNGAT-AE, which achieves collaborative extraction of spatiotemporal features from structural responses by integrating the temporal modeling capability of TCN with spatial correlation mining advantages of GAT. Building upon this model, we establish an unsupervised framework for near-real-time damage detection, comprising two core operational processes: offline training and online detection, as detailed below.

#### 3.2.1. Offline Process: Data Preparation and Model Training

1. Data Acquisition and Preprocessing: Vibration response data is collected through the deployed sensor network while the structure remains in its healthy state. Raw signals undergo preprocessing procedures including noise filtering to eliminate environmental interference and high-frequency noise. The processed data is subsequently partitioned into training and validation sets according to a predetermined ratio, designated for model parameter optimization and performance evaluation respectively.
2. Input Data Construction: Multi-sensor time-series response data are segmented using a sliding window approach, generating data slices $X^{\left(k\right)}\in {\mathbb{R}}^{N\times T}$ (where N represents the number of sensors, T the window length, and k the segment index). Based on physical sensor coordinates, an adjacency matrix is constructed to represent its spatial topology. The adjacency matrix A, representing the spatial topology based on sensor coordinates, is constructed using the Euclidean distance between nodes $v_{i}$ and $v_{j}$. The node relationships are defined as follows:

(8) $$d(v_{i},v_{j})=\sqrt{{\left(x_{vi}-x_{vj}\right)}^{2}+{\left(y_{vi}-y_{vj}\right)}^{2}}$$

(9) $$A_{ij}=\left\{\begin{array}{l} 1,\;\mathrm{if}\;i=j\\ 1,\;\mathrm{if}\;d(v_{i},v_{j})<d_{th}\;\mathrm{and}\;i\neq j\\ 0,\;\mathrm{otherwise} \end{array}\right.$$

where $v_{i}$ and $v_{j}$ denote two sensors, $\left(x_{vi},y_{vi}\right)$ and $\left(x_{vj},y_{vj}\right)$ are their corresponding coordinates respectively; $d(v_{i},v_{j})$ is the Euclidean distance between sensor $v_{i}$ and $v_{j}$;$d_{th}$ is a predefined distance threshold used to determine the adjacency relationship between nodes; and $A_{ij}$ is an element of the adjacency matrix A, where $A_{ij}=1$ indicates an adjacency relationship between $v_{i}$ and $v_{j}$, and $A_{ij}=0$ otherwise; the condition i=j introduces self-loops in the graph, ensuring that each node retains its own features during graph convolution operations [27,39].

Although the graph structure is initially constructed based on sensor proximity, the attention mechanism in GAT allows the model to adaptively learn the importance of connections beyond mere physical distance, thus enhancing its capability to capture damage-sensitive spatial patterns.

3. Model Training and Optimization: The Mean Squared Error (MSE) between input data and reconstructed output serves as the loss function. Parameters of both encoder and decoder are iteratively updated through backpropagation algorithm until the reconstruction error on the validation set demonstrates stable convergence.
4. Damage Threshold Determination: The reconstruction MSE is employed as the Structural Damage Indicator (SDI). To effectively discriminate between intact and damaged structural states, this paper introduces a threshold determination method based on the “3σ criterion”—a well-established empirical threshold in engineering applications suitable for Gaussian-distributed data [40]. It should be noted that this criterion inherently assumes approximate normality of the underlying data. As an optional robustness check, distribution-insensitive quantile-based thresholds (e.g., the 95th percentile) can be considered if significant deviation from normality is observed. The threshold is defined as:

(10) Threshold=μ+3σ

where μ and σ represent the mean and standard deviation, respectively, of the SDI distribution under healthy structural conditions.

#### 3.2.2. Online Process: Near-Real-Time Damage Detection

The online phase utilizes the pre-trained TCNGAT-AE model to perform real-time damage assessment under unknown structural states. The specific procedure consists of:

1. Preprocessing real-time sensor response data through filtering and standardization, followed by segmentation using the identical sliding window strategy to obtain $X^{\left(k^{\prime }\right)}$.
2. Feeding $X^{\left(k^{\prime }\right)}$ to the model and computing the corresponding SDI values. Structural damage is identified when the calculated SDI exceeds the predetermined threshold.

Due to the relatively low computational requirements of SDI calculation, this model achieves near-real-time monitoring by evaluating SDI across consecutive time windows, with one SDI output generated per window. Taking a 200 Hz sampling rate, a window length of 128 samples, and a hop size of 4 samples as an example: on an ordinary laptop (equipped with an Intel^®^ Core™ i7-14700HX CPU and an NVIDIA GeForce RTX 4070 GPU), the time required for data preprocessing and SDI calculation for one time window is less than 0.01 s. The nominal latency includes two key parts: the initial start-up latency is approximately 0.64 s (derived from 128 samples/200 Hz, required to collect the first full window of data), and the subsequent output interval between consecutive SDIs is 0.02 s (derived from 4 samples/200 Hz), ensuring continuous, timely near-real-time tracking of structural states.

**Figure 4:**
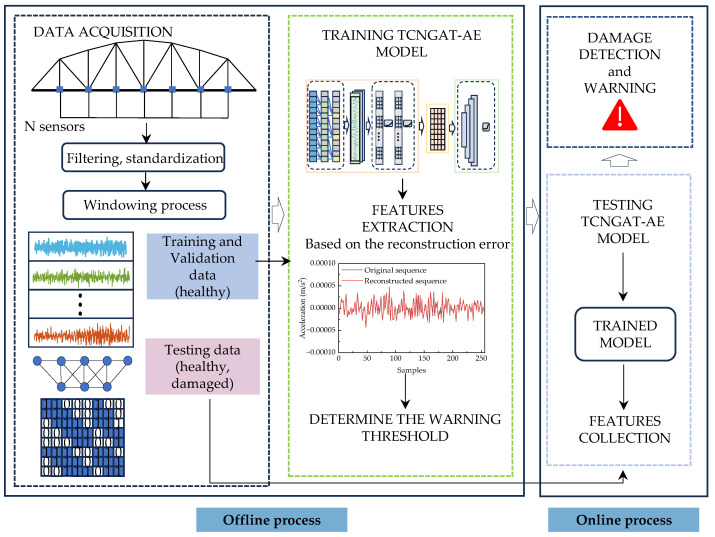
Damage detection process based on TCNGAT-AE.

The complete workflow of the TCNGAT-AE-based near-real-time damage detection methodology is illustrated in Figure 4, which clearly delineates the logical relationships among the three core stages: data acquisition, network training, and near-real-time damage detection.

## 4. Case Study Validation and Discussion

### 4.1. Introduction

This chapter presents the experimental validation of the proposed TCNGAT-AE-based damage detection method. The evaluation employs two real-world bridge case studies: the Z-24 concrete box-girder and the Old Ada steel truss. These cases were selected to establish complementary validation scenarios, encompassing distinct structural typologies, excitation sources, and damage mechanisms. The comparative analysis across these diverse structural systems and operational environments provides a rigorous assessment of the method’s generalization capability. Detailed case studies and a comprehensive performance analysis are presented in the subsequent sections.

### 4.2. Z-24 Bridge Verification

#### 4.2.1. Case Description

**Figure 5:**
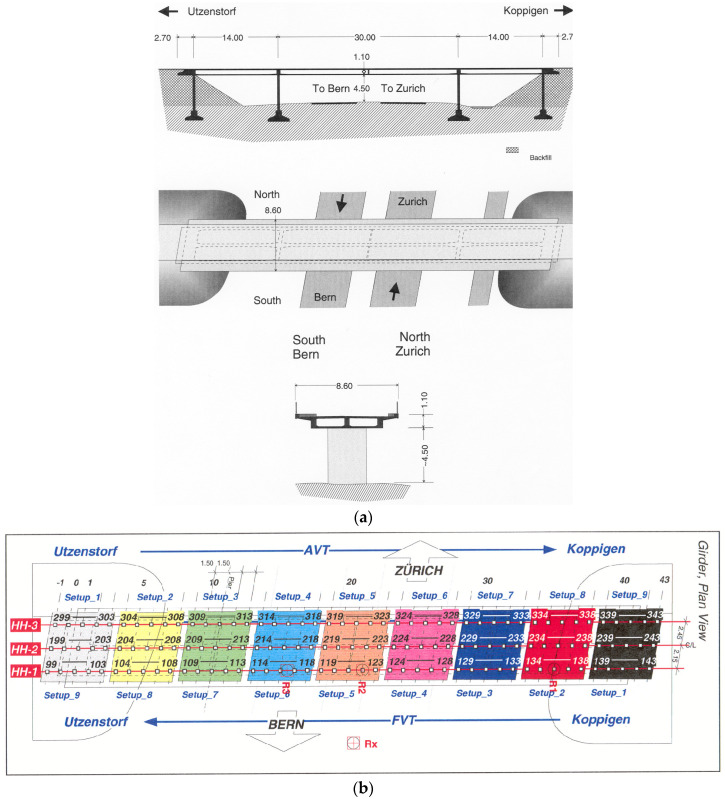
(**a**) Schematic diagram of the Z-24 bridge structure (unit: m); (**b**) Schematic diagram of the sensor layout configuration (unit: m).

The Z-24 bridge, constructed in 1961, is a post-tensioned concrete box-girder structure with a 30-m main span and two 14-m side spans, as schematically illustrated in Figure 5a. Prior to its demolition, the structure underwent a series of progressive damage tests where controlled artificial damage was introduced to capture the structural response evolution during damage progression. The monitoring program encompassed one baseline healthy condition and sixteen damage scenarios. This study focuses on analyzing the first six scenarios, consisting of one healthy state and five damage conditions involving varying degrees of support settlement and foundation inclination, with detailed specifications provided in Table 1. Owing to the limited number of available accelerometers, a sequential testing strategy with nine measurement setups was employed. As shown in Figure 5b, each setup consisted of 15 vertical accelerometers on the bridge deck, where the numbered points (e.g., 99, 103, 104) indicate individual sensor locations. Three fixed reference points (R1, R2, R3) were maintained across all setups. The sampling frequency was set at 100 Hz, with each test recording containing 65,536 acceleration data points [41,42,43].

#### 4.2.2. Experimental Configuration

**Figure 6:**
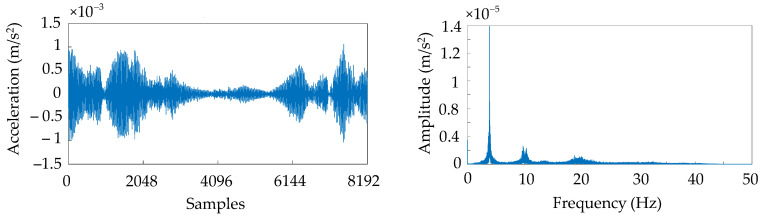
Acceleration time history diagram and corresponding frequency domain diagram.

The window size selection for the Z-24 bridge was optimized to achieve an optimal balance between near-real-time damage detection and feature extraction accuracy, taking into account its environmental excitation characteristics. With a 100 Hz sampling frequency, a 256-point window configuration (equivalent to 2.56 s duration) was established. This temporal length ensures local signal stationarity while satisfying the practical constraints of near-real-time processing latency. Furthermore, it adequately captures the complete multi-mode structural responses characteristic of environmental vibration conditions. The selected window parameters provide a solid foundation for reliable feature extraction and effective model training. Figure 6 presents the acceleration time-history and corresponding frequency spectrum obtained from a representative sensor installed on the Z-24 bridge, illustrating the signal characteristics under this configuration [41].

To support the deep learning model in capturing underlying patterns in vibration signals, sufficient training samples are essential. Using a sliding window approach with a stride of 12, the healthy state data yielded 5441 samples. These were randomly divided into training and validation sets following an 8:2 ratio. The training set comprises 4353 samples (dimensionality: 4353 × 15 × 256) for parameter optimization, while the validation set contains 1088 samples (dimensionality: 1088 × 15 × 256) for generalization assessment and overfitting prevention.

**Figure 7:**
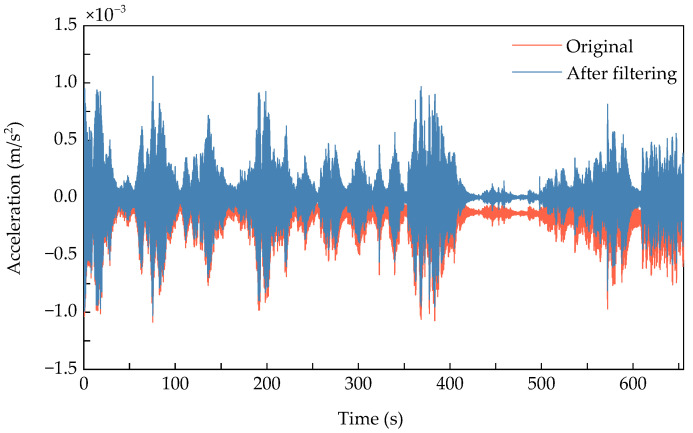
Filtering accelerometer signals.

Environmental factors such as temperature and humidity introduce bias and drift errors in sensor measurements. To address this, Ordinary Least Squares (OLS) regression filtering was applied to eliminate the “systematic error” inherent to each sensor. Figure 7 demonstrates the signal comparison before and after filtering [44]. In this method, the specific size of the fitting window for the linear model varies between the training and test phases of the Z-24 bridge dataset: for the training data, the linear model is fitted using the entire time-series dataset of each sensor (i.e., all training data from a single sensor) to establish a baseline for correcting systematic errors; for the test data, the linear model is fitted independently for each data window (e.g., the same 256-sample window as used in real-time monitoring, consistent with the window configuration applied in subsequent analyses) to adapt to dynamic test conditions. Then, a signal free of drift and bias errors is obtained by subtracting the linear model $\hat{y}$ from the data y as follows:

(11) $$X^{\prime }=y-\hat{y}$$

To normalize amplitude variations across sensors, accelerate training convergence, and enhance model generalization, global Z-score standardization was implemented using training set statistics [45]. The standardization follows:

(12) $$X^{\prime \prime }=\frac{X^{\prime }-\mu_{train}}{\sigma_{train}}$$

where $X^{\prime }$ denotes the data after filtering, $\mu_{train}$ is training set mean, and $\sigma_{train}$ is the training set standard deviation.

Hyperparameter selection critically influences the model’s capacity for spatiotemporal feature extraction. It also governs training stability and generalization performance. In the TCN component, architectural parameters—including layer depth, kernel size, and feature channel count—collectively regulate temporal resolution, receptive field scope, and feature richness. The incorporation of dilated convolutions further enables efficient capture of long-range dependencies while maintaining parameter efficiency. For the GAT module, hidden layer dimensions, architectural depth, attention heads, and dropout rates coordinate to model complex spatial interactions among distributed sensors. The optimization protocol employs adaptive batch sizing and carefully calibrated learning rates to maintain training stability across varying data volumes while ensuring convergence toward optimal solutions. Hyperparameter optimization was conducted through Bayesian optimization implemented via the Optuna framework, a dedicated platform for automated hyperparameter tuning in deep learning applications. The search space was constructed by synthesizing domain-specific expertise in structural health monitoring, architectural constraints of the proposed model, and empirical evidence from preliminary investigations [19,20,21,24,25,26,27,28,29]. The TCN parameter space encompassed: 2–5 network layers, kernel dimensions selected from {3, 5, 8, 16, 32}, and feature channels chosen from {8, 16, 32, 64}. The GAT configuration space included: hidden representations {8, 16, 32, 64}, 1–3 structural layers, attention heads {2, 4}, and dropout regularization between 0.2–0.4 (increments of 0.1). Optimization parameters featured batch sizes {32, 64, 128, 256} and initial learning rates spanning 5 × 10^−4^ to 5 × 10^−3^ (logarithmic scale). The AdamW optimizer was deployed across 30 experimental trials to achieve comprehensive search coverage while maintaining computational efficiency, with all optimization directed toward minimizing the Mean Squared Error (MSE) objective function.

The final parameter configuration is summarized in Table 2. The model contains a total of 1,283,608 trainable parameters, with detailed network architecture provided in Table 3.

**Figure 8:**
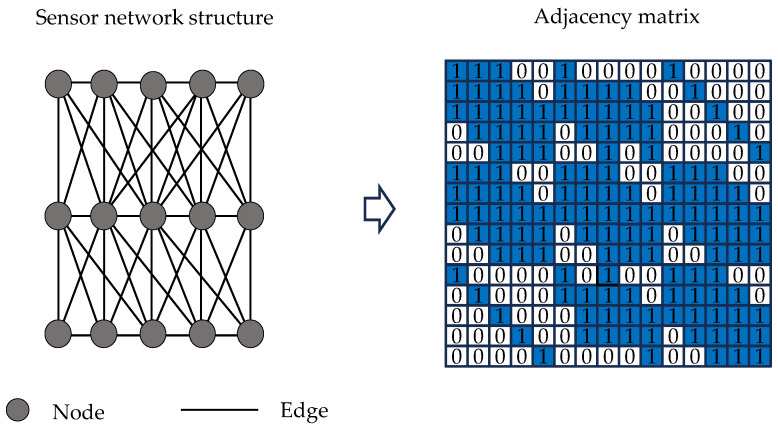
Sensor network graph and adjacency matrix for Z-24 bridge verification.

Following Equations (8) and (9) in Section 3.2, the adjacency matrix representing the spatial topology was constructed using the physical coordinates of the sensor deployment, as presented in Figure 8.

#### 4.2.3. Results and Analysis

**Figure 9:**
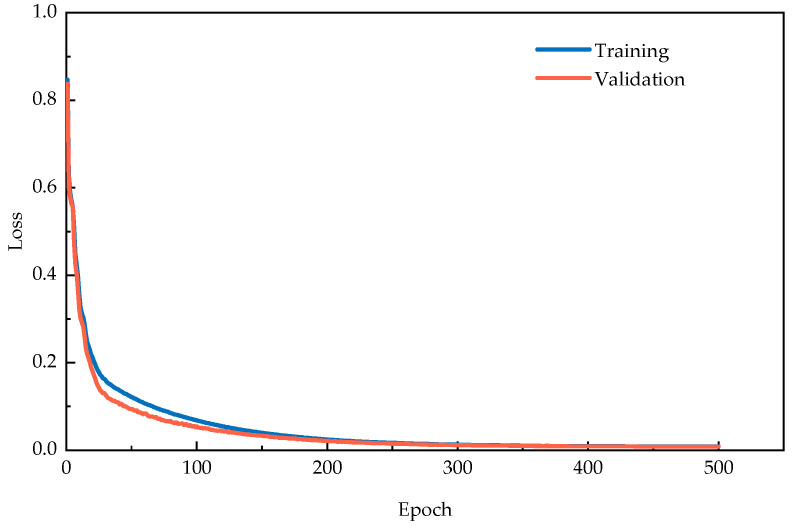
Training and validation loss curves during training process for Z-24 bridge verification.

Figure 9 exhibits the convergence behavior of loss functions throughout the training process. Both training and validation losses demonstrate rapid decay with increasing epochs, while their closely aligned trajectories indicate stable optimization without apparent overfitting.

**Figure 10:**
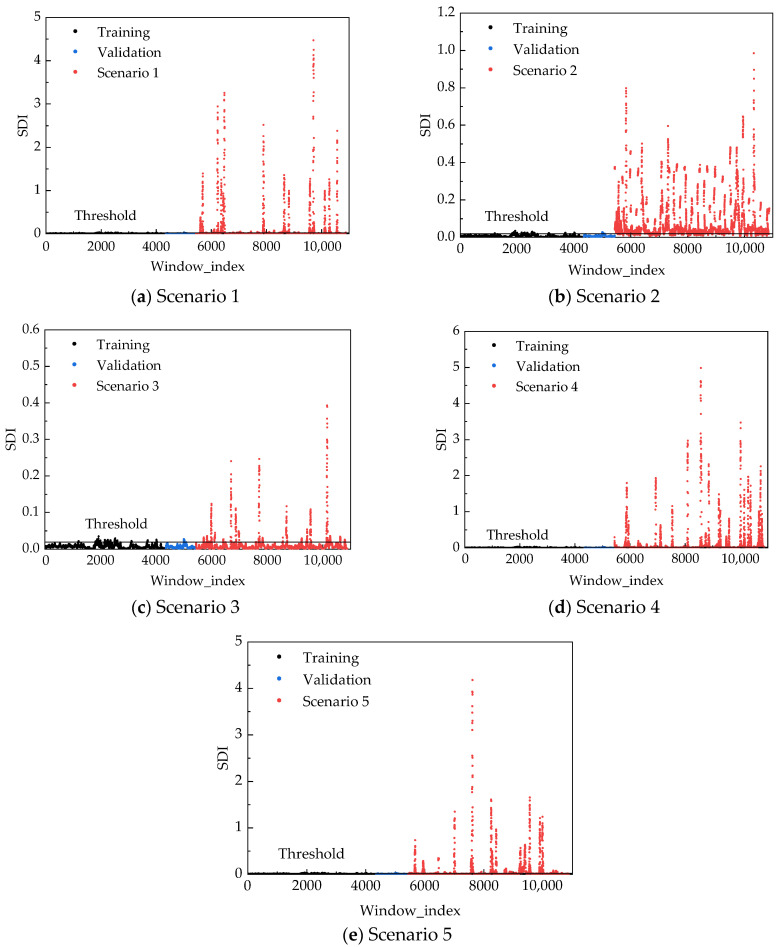
SDI distribution diagrams for each scenario.

Figure 10 depicts the Structural Damage Indicator (SDI) distributions through five subplots corresponding to different damage scenarios. Each subplot illustrates the SDI spread using distinct markers: training set values under healthy conditions (black scatter), validation set values under healthy conditions (blue scatter), and measurements from specific damage scenarios (red scatter). The visualizations reveal pronounced distributional shifts between intact and damaged states.

To quantitatively evaluate the statistical significance of damage-induced effects, two-sample *t*-tests were performed between SDI sequences from each damage scenario and the healthy baseline. The results unequivocally confirm statistically significant deviations (*p* < 0.001) across all damage scenarios, substantially exceeding the standard significance threshold of 0.05. This rigorous statistical evidence validates the sensitivity of the proposed SDI metric to genuine structural damage, excluding the possibility of random fluctuations or model artifacts.

Under healthy structural conditions, the SDI maintains a baseline mean of 0.006120 with a standard deviation of 0.004229, reflecting consistently low values with minimal fluctuations. This stability demonstrates the model’s exceptional reconstruction capability for undamaged states. Establishing a damage threshold at 0.018806 (derived from the healthy validation set’s upper bound), Figure 10 clearly differentiates various damage patterns. Quantitative performance metrics, including scenario-specific means, standard deviations, and threshold exceedance rates, are systematically compiled in Table 4. All damage scenarios exhibit substantially elevated SDI means compared to the healthy reference (0.006), confirming the metric’s fundamental sensitivity to structural alterations. More notably, the anomaly detection rates—defined as the percentage of SDI values surpassing the 0.018806 threshold—reveal marked disparity across damage scenarios. For instance, Scenario 2 shows concentrated high SDI values with 84.03% exceedance rate, whereas Scenario 4 displays broader dispersion (mean: 0.136457, SD: 0.426527). These differential patterns reflect the distinct manifestation of various damage modalities on structural dynamic characteristics, further substantiating the discriminative capacity of the SDI metric through statistical validation.

**Figure 11:**
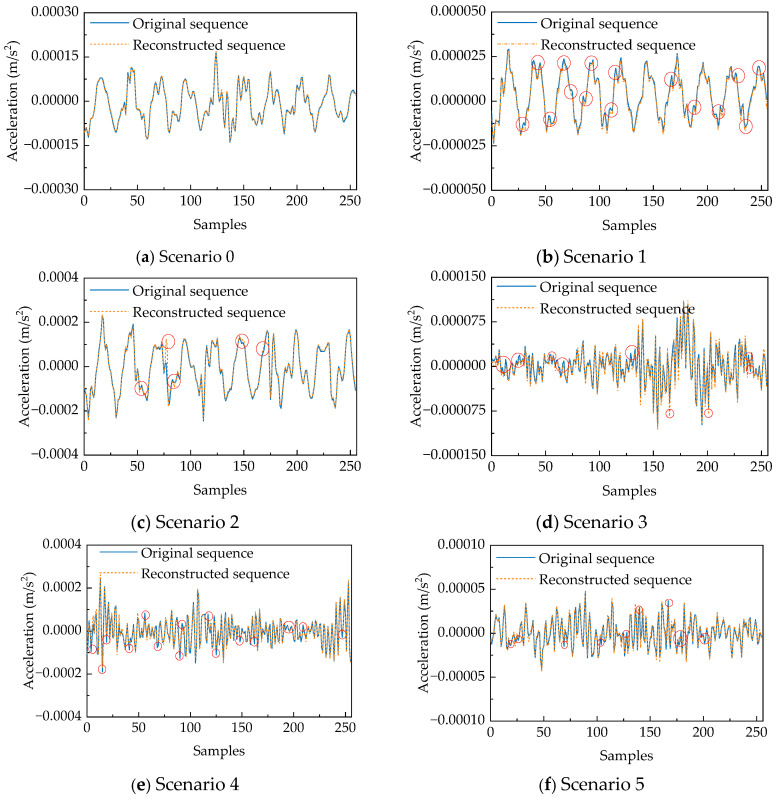
Comparison chart of the original and reconstructed accelerations of the same sensor under different working conditions.

Figure 11 presents a comparative analysis of original and reconstructed signals from an identical sensor under healthy and damaged conditions, using randomly selected time windows. Reconstruction anomalies are explicitly annotated with red circles. Subplot (a) exemplifies reconstruction performance under healthy conditions, where the reconstructed signal maintains high consistency with the original waveform throughout the temporal sequence. The reconstruction demonstrates particular accuracy at critical characteristic points including peaks and troughs, with the reconstructed curve exhibiting precise alignment and overall smoothness. In contrast, damaged conditions reveal substantial reconstruction quality degradation manifesting through distinct anomalous patterns: Subplots (**b**), (**e**), and (**f**) display significant deviations near signal extrema, where the reconstruction fails to accurately capture amplitude variations or rapid transitions. Subplots (**c**) and (**d**) exhibit pronounced waveform distortion accompanied by non-smooth fluctuations. Collectively, reconstructed signals under damaged conditions lose the smooth trajectory characteristics observed in healthy states, developing abrupt inflection points and localized oscillations at critical locations. These systematic reconstruction anomalies demonstrate the model’s sensitivity to abnormal dynamic characteristics and its capability for damage-aware pattern recognition.

#### 4.2.4. Ablation Study

To assess the synergistic interaction between TCN and GAT components within the TCNGAT-AE architecture, we conducted a comprehensive ablation study. This investigation systematically evaluates the efficacy of spatiotemporal feature fusion by comparing performance metrics between single-modality configurations and the integrated approach. The experimental design incorporates two baseline models for reference: (1) TCN-AE, employing solely Temporal Convolutional Networks for temporal feature extraction while ignoring inter-sensor spatial correlations; (2) GAT-AE, utilizing only Graph Attention Networks to capture spatial relationships among sensors without modeling temporal dynamics. All models were configured with identical hyperparameter sets to ensure equitable comparison. The evaluation employed anomaly detection rate as the primary metric, defined as the proportion of time windows where the Structural Damage Indicator (SDI) exceeds the statistically derived threshold based on the “3σ criterion” relative to the total windows in damage phases. This metric effectively quantifies model sensitivity and robustness in damage detection. Comparative analysis of detection performance across multiple damage scenarios elucidates the specific contributions of TCN-GAT integration, thereby providing empirical justification for the unified architecture.

**Figure 12:**
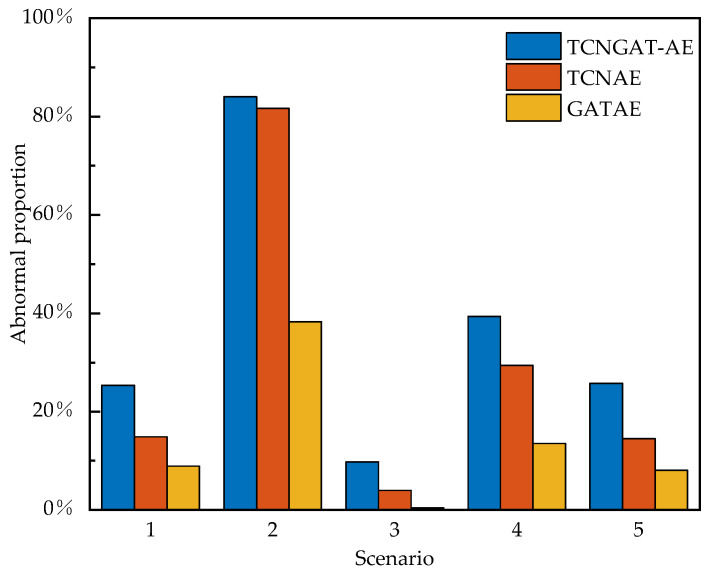
Comparison of abnormal proportions for three models under different scenarios.

As quantitatively demonstrated in Figure 12, the TCNGAT-AE model achieves superior detection performance across all five damage scenarios. The detailed results are as follows: Scenario 1: TCNGAT-AE’s detection rate of 25.37% represents relative improvements of 69.71% over TCN-AE and 183.46% over GAT-AE, respectively; Scenario 2: TCNGAT-AE achieves an 84.03% detection rate, with relative improvements of 2.91% over TCN-AE and 119.34% over GAT-AE; Scenario 3: TCNGAT-AE achieves a 9.80% detection rate, corresponding to relative improvements of 145.61% over TCN-AE and 2030.43% over GAT-AE; Scenario 4: TCNGAT-AE achieves a 39.36% detection rate, with relative improvements of 33.92% over TCN-AE and 190.50% over GAT-AE; Scenario 5: TCNGAT-AE’s detection rate of 25.79% reflects relative improvements of 77.37% over TCN-AE and 218.00% over GAT-AE. The consistent and substantial outperformance of TCNGAT-AE over both single-modality baselines conclusively validates the critical importance of simultaneous spatiotemporal feature learning for optimal damage detection.

### 4.3. Old Ada Bridge Verification

#### 4.3.1. Case Description

**Figure 13:**
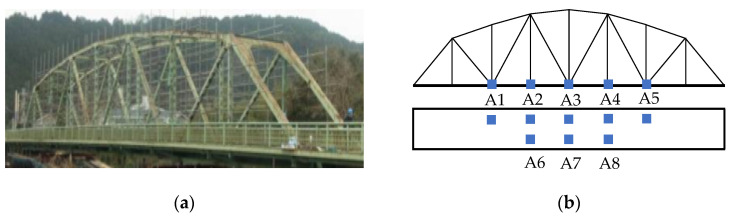
(**a**) Side front view of the Old Ada Bridge; (**b**) Sketch with sensor layout.

**Figure 14:**
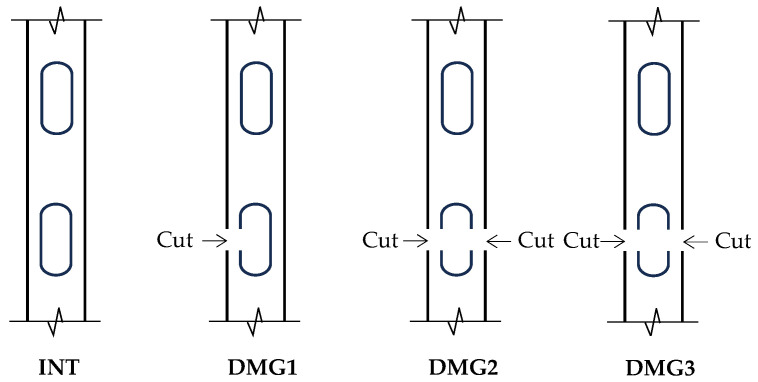
Sketches of each damage case.

The Old Ada Bridge, constructed in 1959, was a steel truss test structure with overall dimensions of 59 m in length, 3.6 m in deck width, and 8 m in structural height. Prior to its demolition in 2012, the bridge served as an experimental platform for vehicle-based structural health monitoring research. The sensor configuration, detailed in Figure 13b, consisted of eight uniaxial accelerometers installed on the bridge deck and operating at a sampling frequency of 200 Hz. Five accelerometers (A1-A5) were mounted in the web member region of one truss, while three sensors (A6-A8) were positioned at corresponding locations on the opposite side [46]. Comprehensive details regarding experimental instrumentation and testing protocols are documented in studies by Chang and Kim [47,48]. Damage scenarios were simulated through controlled cutting of vertical members, with the damage configuration scheme illustrated in Figure 14. The experimental program comprised four test conditions (Table 5), including one baseline intact condition (INT) and three damage conditions (DMG1-DMG3). Each condition involved 10–12 valid tests conducted at vehicle speeds of either 30 km/h or 40 km/h, with damage states introduced through sequential cutting of vertical members as schematically represented.

#### 4.3.2. Experimental Configuration

**Figure 15:**
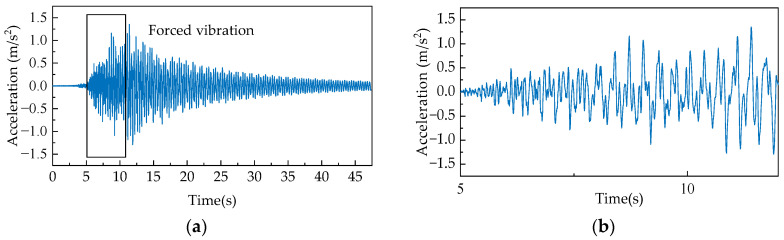
(**a**) Sensor acceleration data; (**b**) Sensor acceleration data during vehicle passage.

Structural damage in bridges fundamentally alters their dynamic mechanical properties, with these modifications being most pronounced under dynamic loading conditions. Therefore, structural responses recorded during vehicle passages were selected as input data, as depicted in Figure 15. To satisfy the requirements of near-real-time damage detection, the window size was optimized to minimize temporal latency while preserving essential signal characteristics, ultimately establishing 128 points as the optimal window length. The model was trained exclusively on data from the healthy state, with the generated windows randomly partitioned into training and validation sets using an 8:2 ratio. To address the challenge of limited sample size, a sliding window approach with a stride of 4 was implemented, resulting in a final training set of 1776 samples with dimensions 1776 × 8 × 128. Global Z-score standardization was applied consistently across all datasets using statistical parameters derived from the training set.

**Figure 16:**
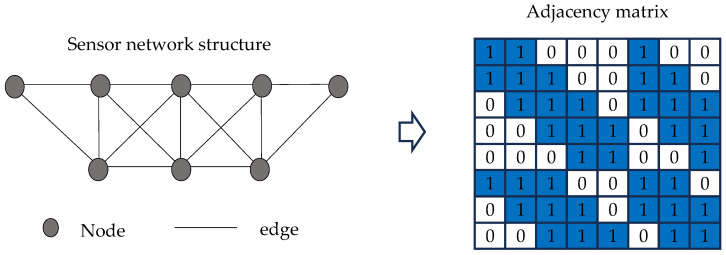
Sensor network graph and adjacency matrix for Old Ada Bridge verification.

Following the methodology detailed in Section 3.2 (Equations (8) and (9)), an adjacency matrix representing the spatial topology was constructed based on physical sensor coordinates, as illustrated in Figure 16. Hyperparameter optimization was performed using the identical Bayesian optimization framework and parameter search space employed in previous experiments, among which the search space for batch sizes was adjusted to {16, 32, 64} considering the limited amount of data. The final optimized parameters are summarized in Table 6. The model architecture, detailed in Table 3, contains 1,422,382 trainable parameters and can be implemented by substituting the specified hyperparameters.

#### 4.3.3. Results and Analysis

**Figure 17:**
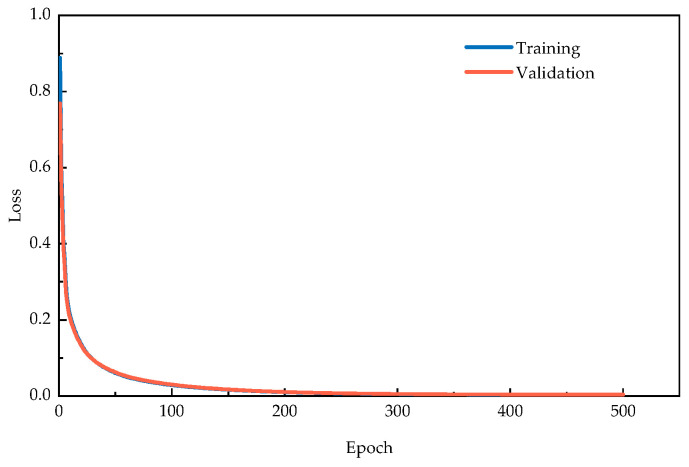
Training and validation loss curves during training process of Old Ada Bridge verification.

The evolution of training and validation losses across training epochs is presented in Figure 17. The curves exhibit a sharp reduction during initial training phases before progressively stabilizing, demonstrating effective parameter optimization. The consistent convergence between training and validation metrics indicates robust generalization capability without overfitting.

**Figure 18:**
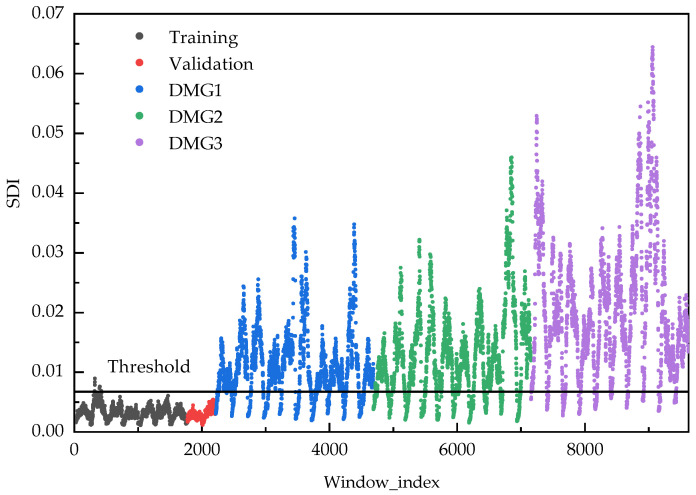
SDI distribution under different cases.

Figure 18 displays the temporal evolution of the Structural Damage Indicator (SDI) across three damage scenarios. Statistical analysis using two-sample *t*-tests reveals significant differences (*p* < 0.001) between SDI distributions in damaged and healthy states, unequivocally confirming that the observed variations originate from structural damage rather than random fluctuations. In the healthy state, the SDI distribution demonstrates a mean of 0.002867 and standard deviation of 0.000849, establishing a damage threshold of 0.005413 (mean + 3σ). All damage scenarios show substantially elevated SDI means (DMG1: 0.00694; DMG2: 0.012704; DMG3: 0.020267) with increased standard deviations, indicating enhanced response variability under damaged conditions and validating the method’s sensitivity to damage presence and severity progression.

**Figure 19:**
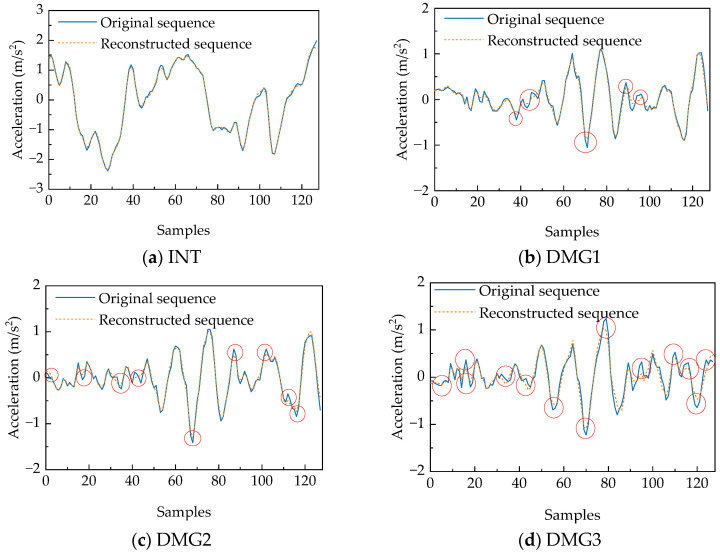
Comparison chart of the original and reconstructed accelerations of the same sensor under different case.

Signal reconstruction performance across different conditions is illustrated in Figure 19. Under intact conditions (INT), the representative window achieves an MSE of 0.002589 with excellent reconstruction fidelity in waveform morphology, amplitude characteristics, and phase alignment. Damage scenarios exhibit progressively elevated MSE values (DMG1: 0.004319; DMG2: 0.004663; DMG3: 0.011997), with reconstruction errors predominantly localized at response extrema (peaks and troughs) as marked by red circles. The observed amplitude attenuation and phase distortion become increasingly pronounced with damage severity, demonstrating that structural damage alters dynamic characteristics and consequently degrades reconstruction capability. These findings validate the effectiveness of reconstruction-error-based damage assessment.

#### 4.3.4. Ablation Study

To comprehensively assess the damage detection performance of the TCNGAT-AE framework under vehicular loading conditions, we conducted rigorous ablation experiments utilizing monitoring data from the Old Ada Bridge, with systematic comparisons against two baseline architectures (TCN-AE and GAT-AE).

**Figure 20:**
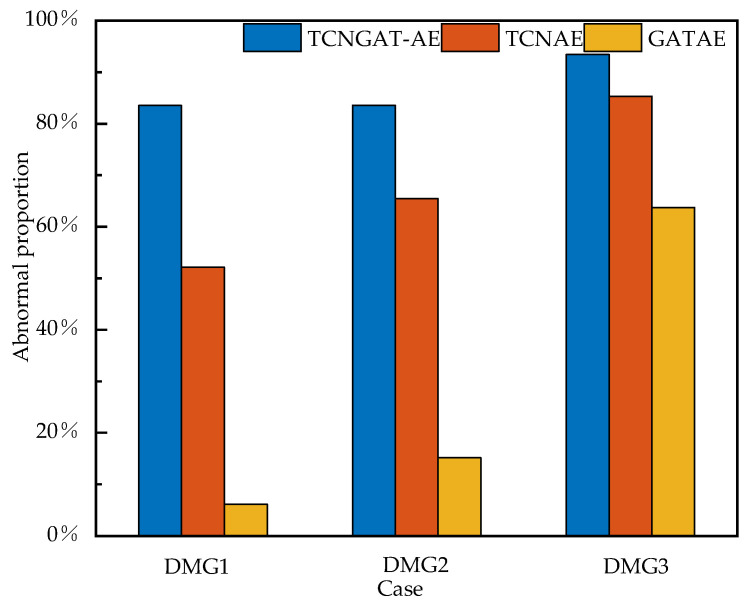
Comparison of abnormal proportions for three models under different cases.

As demonstrated in Figure 20, TCNGAT-AE maintained consistent performance superiority across all damage scenarios. The model achieved an 83.53% anomaly detection rate under DMG1 conditions, representing significant improvements over TCN-AE (52.14%) and GAT-AE (6.16%). In the DMG2 scenario, it attained an 83.56% detection rate, exceeding both reference models (65.45% and 15.19%). For the most severe DMG3 condition, the framework reached a 93.44% detection rate, again outperforming both benchmarks (85.34% and 63.75%). Particularly notable is the 126% relative performance enhancement over GAT-AE in detecting minor damage (DMG1), highlighting the integrated architecture’s exceptional sensitivity to incipient structural deterioration. Experimental analysis further reveals the predominant importance of temporal characteristics under transient vehicular excitation, as evidenced by TCN-AE’s consistent advantage over GAT-AE. Nevertheless, through effective spatiotemporal feature integration, TCNGAT-AE achieved additional performance gains of 60.2%, 27.6%, and 9.5% over TCN-AE for DMG1 through DMG3 scenarios, respectively, unequivocally validating the critical contribution of spatial correlation modeling to damage detection capability.

These findings substantiate that TCNGAT-AE’s synergistic integration of temporal dynamics and spatial topological relationships facilitates more comprehensive and robust damage assessment in vehicle load environments. This conclusion reinforces previous experimental outcomes from the Z-24 Bridge under ambient excitation, collectively affirming the efficacy and generalization capacity of the proposed spatiotemporal fusion methodology across diverse loading conditions and structural configurations.

### 4.4. Comprehensive Analysis and Discussion

Validation results from both the Z-24 concrete box-girder bridge and the Old Ada Bridge (a steel truss bridge) demonstrate that the TCNGAT-AE-based damage detection method exhibits notable cross-structural generalization capability. The substantial differences between these two bridges in terms of structural typology, excitation sources, and damage mechanisms create an ideal testbed for evaluating methodological adaptability. Experimental findings reveal consistent damage detection performance across different structural systems, suggesting that the method learns fundamental spatiotemporal dynamic characteristics of structural health states rather than merely memorizing surface-level response patterns of specific structures. When damage occurs, the model effectively captures subtle variations in spatiotemporal features and manifests corresponding sensitivity through reconstruction error metrics.

Architectural analysis through ablation studies indicates that the comparatively weaker performance of the GAT-AE model may be attributed to its framework characteristic of processing raw vibration signals directly. Environmental noise and complex fluctuations inherent in raw signals potentially compromise the attention mechanism’s capacity for accurate spatial correlation modeling. In contrast, TCNGAT-AE employs a cascaded “temporal-feature-extraction to spatial-correlation-modeling” architecture where the TCN module first extracts representative high-level temporal features from raw signals. This process maintains essential damage information while mitigating noise interference, thereby establishing a more reliable feature foundation for subsequent GAT module operations. This hierarchical processing mechanism represents a significant factor in performance enhancement, with ablation results in Figure 12 providing supporting evidence for spatiotemporal feature fusion effectiveness.

The non-monotonic response pattern observed in the main sensor network’s damage detection results from the Z-24 bridge experiment merits particular attention. The anomaly detection rates for the four damage scenarios measure 25.37% (Scenario 1), 84.03% (Scenario 2), 9.80% (Scenario 3), and 39.36% (Scenario 4), with Scenario 3 showing markedly lower performance. Control experiments using supplementary measurement points reveal a relatively stable increasing trend in detection rates across the same scenarios (7.81%, 12.44%, 12.98%, 17.56%). This discrepancy may indicate substantial variations in damage sensitivity among different sensor locations, where Scenario 3’s primary dynamic response alterations might be constrained by sensor placement configuration. Additionally, the global reconstruction error-based SDI potentially possesses inherent limitations in characterizing localized damage features. These findings highlight the necessity for integrated optimization between sensor network design and damage detection methodologies in practical engineering applications to enhance overall monitoring system reliability.

In summary, the TCNGAT-AE-based damage detection method demonstrates substantial potential for engineering applications. The unsupervised nature of the learning approach effectively addresses the challenge of scarce damage samples in practical scenarios, while the hierarchical spatiotemporal feature processing architecture ensures methodological robustness. Future research should emphasize the development of damage-sensitivity-driven sensor placement theories and the creation of multi-scale damage indicators capable of simultaneously characterizing global properties and local variations, thereby further improving methodological applicability and reliability in complex engineering environments.

## 5. Conclusions

This study presents a novel TCNGAT-AE framework that integrates Temporal Convolutional Networks and Graph Attention Networks to address the critical challenge of spatiotemporal feature extraction in structural health monitoring. The proposed method demonstrates significant advantages through comprehensive validation using field monitoring data from the Z-24 Bridge under environmental excitation and the Old Ada Bridge under vehicular loads. The principal findings are summarized as follows:

- Advanced Spatiotemporal Modeling Architecture
  The hierarchical framework successfully overcomes limitations of conventional methods in capturing spatiotemporal interactions through its unique “temporal-to-spatial” processing paradigm. The TCN module extracts multi-scale temporal patterns while the GAT module captures complex spatial dependencies within the sensor network. Bayesian-optimized hyperparameters enable exceptional signal reconstruction performance, with SDI values of 0.006120 ± 0.004229 and 0.002867 ± 0.000849 for healthy states of Z-24 and Ada bridges respectively. The precise reconstruction at critical waveform characteristics confirms the model’s robust representation capability across varying excitation conditions.
- Substantial Performance Enhancement through Feature Fusion
  Ablation studies on the Z-24 Bridge reveal the crucial importance of integrated spatiotemporal modeling. TCNGAT-AE achieves consistent superiority over single-modality benchmarks, particularly in Scenario 3 (minor damage) where it demonstrates a remarkable 9.80% detection rate—representing improvements of 145.61% and 2030.43% over TCN-AE and GAT-AE respectively. These results unequivocally demonstrate that the synergistic combination of temporal and spatial features significantly enhances damage sensitivity and detection reliability.
- Practical Framework with Demonstrated Generalization Capability
  The implemented “offline-online” operational framework enables near-real-time damage assessment through sliding window processing and statistical thresholding. This end-to-end pipeline processes raw vibration signals directly, eliminating complex preprocessing requirements. Comprehensive testing across both bridges shows significant SDI elevations in all damage scenarios, with statistical significance (*p* < 0.001) confirming damage-induced variations rather than random fluctuations. The framework’s robust performance across diverse structural types and loading conditions underscores its practical engineering value.

## 6. Future Research Directions

While the current study establishes a solid foundation, several promising directions warrant further investigation:

- Dynamic Graph Structure Optimization
  The present approach, though incorporating dynamic attention weights, relies on predefined adjacency matrices based on physical sensor locations. Future work should develop adaptive graph generation mechanisms that continuously update topological connections according to real-time structural response characteristics, enabling more accurate tracking of damage-induced spatial correlation changes.
- Physics-Informed Learning Integration
  Based on physical principles, the finite element method (FEM) is a classical approach for structural state assessment. It offers clear interpretability and uses low-dimensional parameters, making it suitable for scenarios with well-defined properties—such as during the design stage—where it achieves high-fidelity response reconstruction. However, its application to in-service structures faces limitations: performance degrades due to model-reality discrepancies (e.g., hidden cracks or material degradation); strong nonlinear responses often require simplifying assumptions, losing damage-related information; and high-fidelity simulations are computationally expensive, limiting real-time use. Data-driven machine learning methods address these shortcomings. They learn directly from monitoring data (e.g., vibration, strain) without relying on explicit physical models, adapt well to uncertainties, and capture complex nonlinear patterns for accurate damage identification. Once trained offline, they enable millisecond-level inference, meeting real-time engineering needs. Notably, both approaches are complementary. Integrating them—e.g., via Physics-Informed Neural Networks (PINNs)—embeds physical constraints such as equilibrium equations into the learning process, often through physics-based penalty terms in the loss function. This preserves data-driven flexibility while ensuring physical consistency, improving both reliability and applicability in practical structural state assessment [49].
- Application to Building Structures
  The TCNGAT-AE framework demonstrates strong potential for application in building structural health monitoring (SHM). In future work, the framework will be extended to monitor building systems equipped with sensor networks, leveraging the monitoring data characteristics of building structures described in the existing literature [50,51,52,53]. This extension will include evaluating the framework’s performance across diverse structural configurations and validating its generalization capability in spatiotemporal response analysis of multi-sensor systems. By capitalizing on its inherent ability to model spatiotemporal dependencies, the adapted framework will effectively capture the unique dynamic behaviors of building environments, thereby reinforcing its cross-domain applicability and increasing its practical value in real-world structural monitoring.

The TCNGAT-AE framework represents a significant step forward in structural damage detection, with demonstrated capabilities that bridge the gap between theoretical innovation and practical implementation. The identified research directions provide a roadmap for continued advancement in intelligent structural health monitoring systems.

## Figures and Tables

**Table 1:** Details of operating conditions.

Case	Scenario Name	Reversible Damage
Healthy	0	Baseline structure
Damage	1	Support Settlement: 20 mm
	2	Support Settlement: 40 mm
	3	Support Settlement: 80 mm
	4	Support Settlement: 95 mm
	5	Inclination of foundation

**Table 2:** Parameter Configuration for Z-24 bridge verification.

Parameter Name	Value
TCN_layers	3
TCN_kernel	32
TCN_channel	32
GAT_layers	2
Attention_heads	4
GAT_hidden_dim1	32
GAT_hidden_dim2	16
GAT_dropout	0.4
Batch_sizes	128
Init_lr	4.27 × 10^−3^

**Table 3:** Detailed Model Architecture.

Name of Part	Layer	Activation	Input Size	Output Size
Input	Input Layer	-	[128, 15, 256]	[1920, 1, 256]
Encoder Module	TCNBlock1	ReLU	[1920, 1, 256]	[1920, 32, 256]
	TCNBlock2	ReLU	[1920, 32, 256]	[1920, 32, 256]
	TCNBlock3	ReLU	[1920, 32, 256]	[1920, 32, 256]
	Flatten	-	[1920, 32, 256]	[1920, 8192]
	Dense	-	[1920, 8192]	[1920, 128]
	Reshape	-	[1920, 128]	[128, 15, 128]
	GAT Layer1	LeakyReLU + ELU	[128, 15, 128]	[128, 15, 128]
	GAT Layer2	LeakyReLU + ELU	[128, 15, 128]	[128, 15, 64]
Bottleneck Module	Flatten	-	[128, 15, 64]	[128, 960]
	Dense	ReLU	[128, 960]	[128, 64]
	Reshape	-	[128, 64]	[128, 960]
Decoder Module	Conv1D Transpose	ReLU	[128, 15, 64]	[128, 15, 128]
	Conv1D Transpose	-	[128, 15, 128]	[128, 15, 256]

**Table 4:** The average values, standard deviations, and abnormal proportion of SDI in different scenarios.

Scenario Name	Mean	Standard Deviation	Abnormal Proportion
0	0.006120	0.004229	-
1	0.095805	0.367091	25.37%
2	0.085315	0.113103	84.03%
3	0.010968	0.025168	9.80%
4	0.136457	0.426527	39.36%
5	0.062490	0.237510	25.79%

**Table 5:** Description and number of tests for each case.

Case	Description	Vehicle Speed (km/h)	Number of Tests
INT	Intact bridge	30	11
		40	10
		50	5
DMG1	Half-cut in vertical member at midspan	40	12
DMG2	Full-cut in vertical member at midspan	40	10
DMG3	Full-cut in vertical member at 5/8th span	40	10

**Table 6:** Parameter configuration for Old Ada Bridge verification.

Parameter Name	Value
TCN_layers	3
TCN_kernel	32
TCN_channels	64
GAT_layers	2
Attention_heads	4
GAT_hidden_dim1	64
GAT_hidden_dim2	8
GAT_dropout	0.3
Batch_sizes	32
Init_lr	5.68 × 10^−3^

## Data Availability

Data will be available on reasonable request.
